# Power analysis data set for 4-Bit MOCLA adder

**DOI:** 10.1016/j.dib.2017.11.017

**Published:** 2017-11-11

**Authors:** K. Nehru

**Affiliations:** Institute of Aeronautical Engineering, Hyderabad 500043, India

**Keywords:** Low power, Carry look ahead adder, T-spice, Gate diffusion input technique, Binary coded decimal

## Abstract

In order to reduce the silicon area of the chip and optimize the power of arithmetic circuits, this paper proposes a low power carry look-ahead BCD (Binary Coded Decimal) adder which uses a four bit MOCLA (Multiplexer and Or gate based Carry Look Ahead Adder) that forms the basic building block. This proposed MOCLA style uses a 2 input MUX, OR gate and GDI (Gate Diffusion Input) based full adder and PG units and it is used for achieving low power in BCD adder circuits.

**Specifications Table**

**Table:** 

Subject area	*Electronics*
More specific subject area	*Low Power VLSI*
Type of data	*Table, figure*
How data was acquired	*Cadence software virtuoso schematic editor, T spice and layout editor have been applied to attain the data results*
Data format	*Analyzed*
Experimental factors	*Low power MOCLA adder has been proposed. It has been tested with BCD adder circuit*
Experimental features	*Simulation study and layout design has been used to determine results*
Data source location	*India, Hyderabad*
Data accessibility	*Data is available within this article*

**Value of the data**

• Adders are the basic building block to design arithmetic systems. A new low power MOCLA adder has been used to enhance the performance of arithmetic systems.
• The proposed a low power and area efficient low power MOCLA adder including full adder, propagate generate gate, multiplexer and logic OR gates.
• MOCLA adder circuits are widely examined since their performance directly affects the binary coded decimal circuits.
• The presented circuit techniques and power analysis can support the researchers to reduce the foot print of the chip and implement low power arithmetic systems.

## 1 Data

**Fig. 1:**
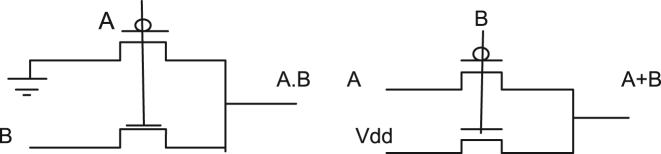
Logic gates using minimum number of MOSFETs [8].

**Fig. 2:**
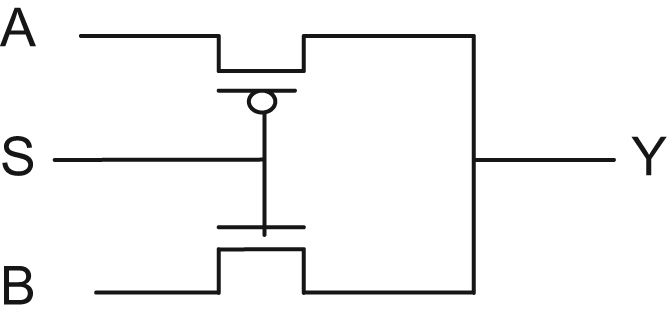
Multiplexer realization using MOSFETs.

**Fig. 3:**
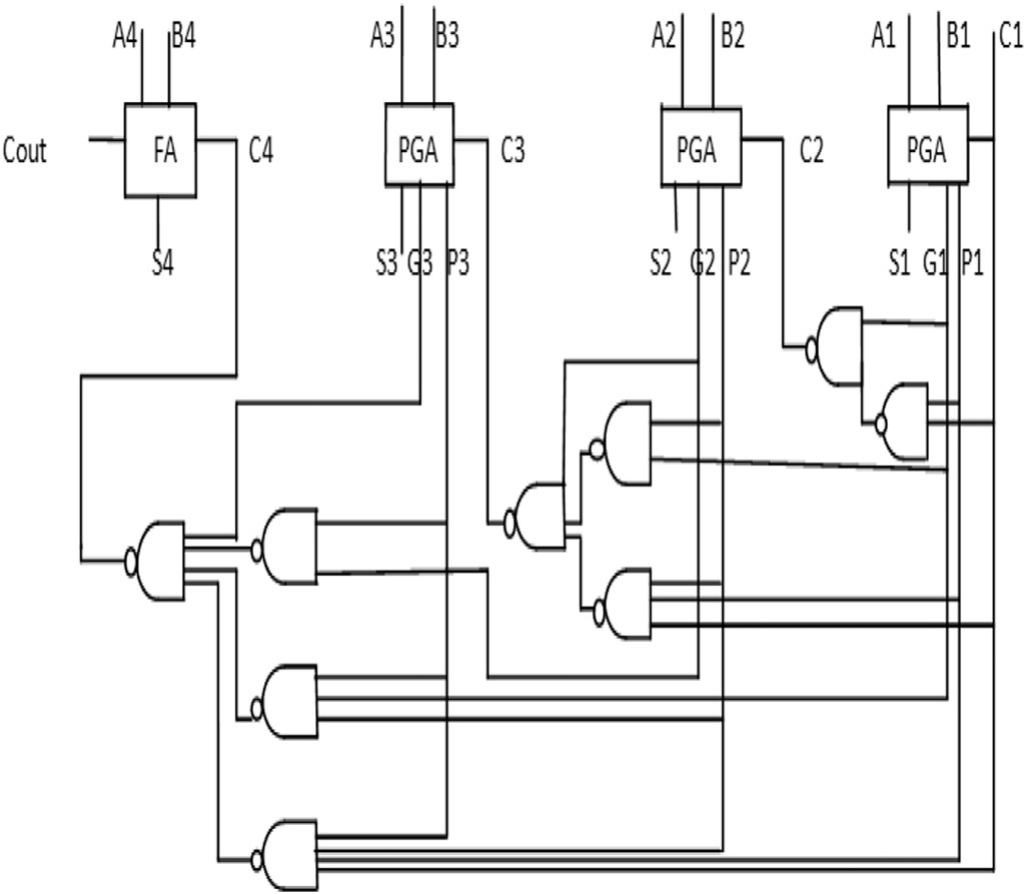
4-Bit MCLA adder architecture using NAND, FA and PGA blocks [5], [6].

**Fig. 4:**
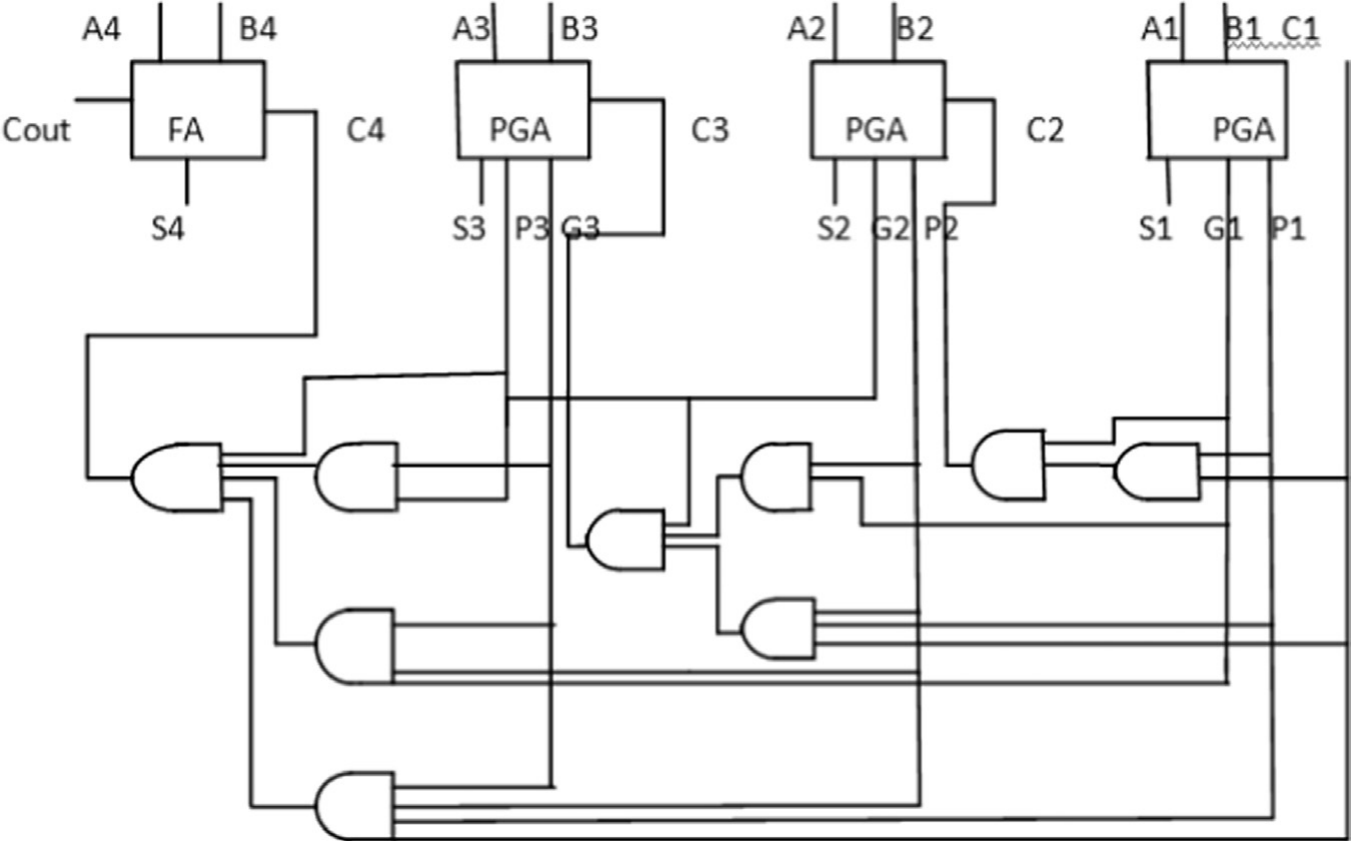
Conventional 4-Bit NCLA [5], [6].

**Fig. 5:**
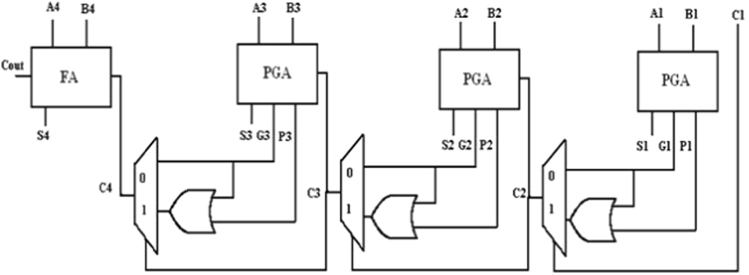
Proposed 4-Bit MOCLA adder architecture.

In this paper, a low power 4-bit adder called the MOCLA adder has been proposed. This MOCLA BCD adder uses the novel two transistor logic gates as shown in Fig. 1. The multiplexer in this proposed logic style of MOCLA to produce the carry output as shown in Fig. 2. In Fig. 3. Shows the MCLA full adder design. This architecture uses the NAND gates instead of the simple AND, OR and NOT gates that are used in the normal adder circuits. The NCLA adder architecture as shown in the Fig. 4. The proposed MOCLA adder architecture uses the combination of the multiplexer and the OR gates to implement the design. Fig. 5. Shows the general block diagram of the proposed MOCLA adder architecture. The evolution of the MOCLA adder has been compared with conventional CLA adders [5], [6]. The detailed comparison results of CLA adders are shown in Table 1.The binary coded decimal adders are designed with the help of carry skip adder for improving speed in decimal arithmetic logic unit [1], [2], [3], [4], [5], [6]. The two input multiplexers used in this adder were realized using only two transistors for area reduction [7]. The conventional carry look adder consist of AND & OR logic gates. MCLA adder's address the problems of delay in carry look-ahead adders by are replaced those by NAND gates for better improvement in delay and area [8]. The proposed a new technique called as GDI technique. The reduction in energy consumption is important in VLSI circuits [9], [10], [11].

**Table 1:** Data set analysis for transistor count and power consumption for adders.

**Logic Styles**	**MOSFETs**	**Average Power Consumption (W)**
Conventional MCLA adder[6]	104	11.651×10^-8^
Conventional NCLA adder [6]	94	2.989890×10^-8^
**Proposed MOCLA adder**	52	0.1236×10^-8^

## 2 Experimental design, materials and methods

### 2.1 Analysis of power consumption

All the simulation results are measured with data patterns at room temperature of 300 K. The maximum frequency rate is 25 MHz. The complete data set of power analysis is done by dataset values of voltage 0.9 at room temperature.The average power consumption and delay of all the techniques in the low power full adder circuits are measured by using transient analysis. The switching power is calculated with an input pattern using Monte Carlo simulation for every clock pulse of the circuit. The propagation delay is estimated as the average sum of the delay occurs in switching transition from 0 to 1 and 1 to 0.The power delay product is calculated by using the values of power and delay of the appropriate circuit. The average power consumption of 4 bit MCLA adder is found to be 74% more power consumption than conventional adder was reported in [6]. The proposed adder has significant improvement in average power consumption over MCLA and NCLA adders. Table 1 shows the power consumption and transistor counts of proposed design and conventional designs. The proposed adder requires the lowest count of transistor as 52 when compared to the other of MCLA and NCLA as 104 and 94 respectively, thus decreasing the area size, power consumption and delay in turn improving the speed of design.
