# Supplementary material for: Power analysis data set for 4-Bit MOCLA adder
Source: Data Brief. 2017 Nov 11;16:122–6. doi: 10.1016/j.dib.2017.11.017 (PMC5699873; doi:10.1016/j.dib.2017.11.017)
Supplement: Supplementary file 1 — Supplementary material [file mmc1.doc]

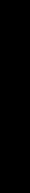
 ***Conflicts of Interest Statement***

***There is no conflict of interest to publish my research article***

**Manuscript title: *Power Analysis Data Set for 4-Bit MOCLA Adder***

The authors whose names are listed immediately below certify that they have NO affiliations with or involvement in any organization or entity with any financial interest (such as honoraria; educational grants; participation in speakers’ bureaus; membership, employment, consultancies, stock ownership, or other equity interest; and expert testimony or patent-licensing arrangements), or non-financial interest (such as personal or professional relationships, affiliations, knowledge or beliefs) in the subject matter or materials discussed in this manuscript.

**Author names: K.NEHRU**
